# Richness and Composition of Mycorrhizal Fungi Varies by Flood Level and River Basin in Oligotrophic Amazonian Seasonally Flooded Forests

**DOI:** 10.1002/ece3.73373

**Published:** 2026-04-08

**Authors:** Maihyra Marina Pombo, Camila Duarte Ritter, Florian Wittmann, Jadson José Souza de Oliveira, Maria Teresa Fernandez Piedade, Jochen Schongart, Alexander Zizka

**Affiliations:** ^1^ Ecology, Monitoring and Sustainable Use of Wetlands Group, Biodiversity Coordination National Institute of Amazonian Research (INPA) Manaus Brazil; ^2^ Juruá Institute Manaus Amazonas Brazil; ^3^ National Institute of Amazonian Research (INPA) Manaus Brazil; ^4^ Department of the Floodplain Institute, Karlsruhe Institute of Technology (KIT) Institute of Geography and Geoecology Rastatt Karlsruhe Germany; ^5^ Wood Pathology Lab., Technology and Innovation Coordination (COTEI) National Institute of Amazonian Research (INPA) Manaus Amazonas Brazil; ^6^ Biodiversity of Plants Lab, Department of Biology University of Marburg Marburg Germany

**Keywords:** *I* *gapó* forest, inundation, metabarcoding, Negro River, root‐fungi interaction, tropical forest

## Abstract

Mycorrhizal fungi play a key role in supporting plants in nutrient‐poor environments, yet their diversity and distribution in Amazonian floodplain forests remain poorly understood. Here, we assessed the presence of mycorrhizal fungi across three seasonally flooded by black water forest (*igapó*) sites in Central Amazonia (RDS Uatumã, RDS Rio Negro, and PARNA Jaú) using DNA metabarcoding of roots. We examined how flood regimes and locality influence the richness and composition of mycorrhizal fungi and, particularly, for ectomycorrhizal fungi (ECM). We found 568 mycorrhizal ASVs, including 307 ECM and 238 arbuscular mycorrhizal fungi. Mycorrhizal richness was higher in sites that experienced prolonged inundation. However, ECM richness was more affected by site than flood level. Community composition varied across sites and flood levels, with distinct fungal assemblages associated with different host tree species. Our results suggest that a complex interplay of flood dynamics, local characteristics, and plant‐fungal interactions shapes the structure of mycorrhizal communities in *igapós*. Despite seasonal flooding, these forests harbor a diverse and specialized mycorrhizal biota, including many ECM lineages rarely documented in tropical wetlands. This study contributes with novel insights into Amazonian belowground biodiversity and highlights the importance of topographic and hydrological heterogeneity in maintaining adapted fungal diversity and function. Understanding these patterns is critical for forecasting the ecological impacts of climate‐driven changes to Amazonian flood regimes.

## Graphical abstract

Conceptual representation of topographic gradients in Amazonian *igapó* forests. High, medium, and low igapós differ in elevation and flood duration, with low igapós experiencing the longest periods of inundation. Fungal mycelia and DNA symbols indicate sampling of root‐associated mycorrhizal communities across the gradient, used to assess the influence of flooding on fungal diversity.

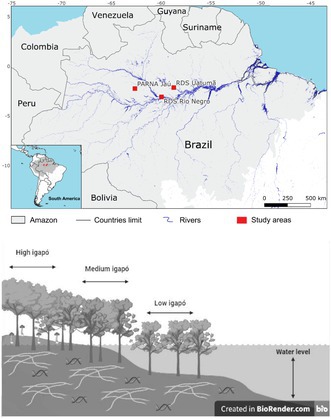

## 1 Introduction

Plants form symbiotic relationships with fungi (mycorrhizae), increasing root nutrient uptake and improving drought resistance of individuals, and thereby the resilience of forest communities. In seasonally flooded environments, however, oxygen deprivation in the soil can lead to shifts in fungal diversity and taxonomic composition disrupting mycorrhizae associations (Das et al. 2025; Martínez‐Arias et al. 2022). In temperate ecosystems, flooding affects mycorrhizal activity and selects for flood‐tolerant taxa (Cho et al. 2021; Thomas 2021); however, these dynamics remain poorly understood in tropical forests. A better understanding of these dynamics is essential to predict forest responses to climate changes and develop conservation strategies, given the crucial role of mycorrhizae in nutrient‐poor tropical soils and the projected increase in extreme flooding events due to climate change (Martínez‐Arias et al. 2022).

**FIGURE 1:**
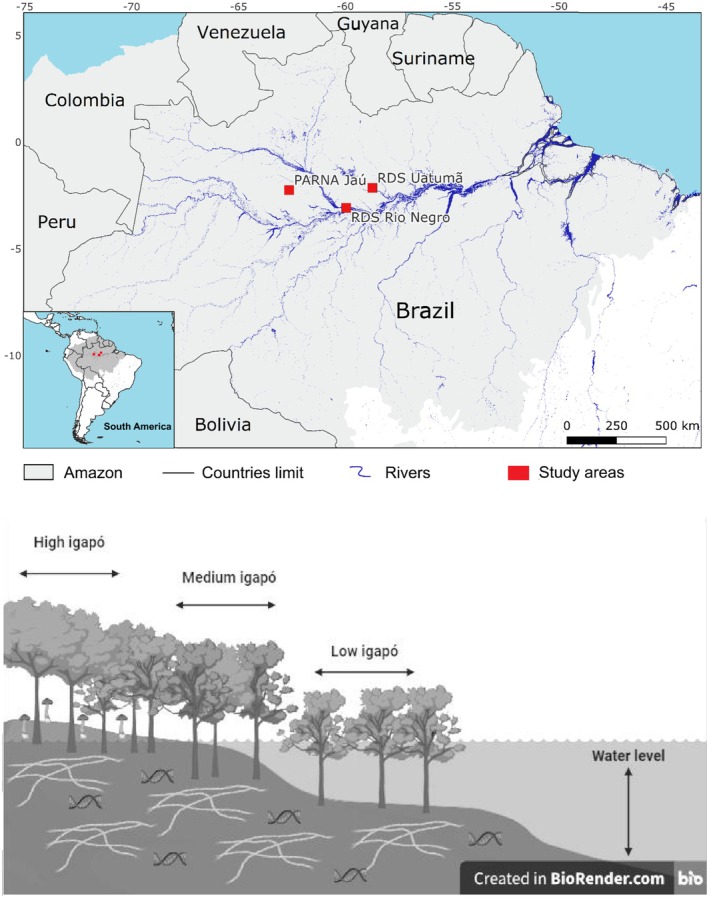
Location of the three study sites in the Brazilian Amazon: PARNA Jaú, RDS Uatumã, and RDS Rio Negro (red squares). The map shows major rivers (blue lines), national boundaries, and the extent of the Amazon biome (light gray). The inset map highlights the location of the study region within South America. Below is a schematic view of the topography of *igapós*. Low *igapós* are flooded for longer, supposedly having a more specialized biota adapted to stress by reduced oxygen, while high *igapós* are less stressed due to a shorter period.

In the major Amazonian rivers, the South American Monsoon System (Marengo et al. 2011) drives seasonal fluctuations in water and sediment discharge, creating an annual “flood pulse” that sustains the world's largest expanse of seasonally flooded forests (Junk et al. 1989). Part of these forests are inundated by nutrient‐poor black waters, known in Brazil as *igapós*, which cover approximately 302,000 km^2^ of the Amazon Basin (Junk et al. 2011, 2024; Wittmann et al. 2022). A characteristic example of these ecosystems is the Rio Negro basin, where around 125,000 km^2^ of predominantly woody vegetation are seasonally flooded (Hess et al. 2015). Flooding follows a marked topographic gradient, with lower areas remaining submerged for longer periods (low *igapó*), intermediate zones experiencing moderate inundation (medium *igapó*), and higher areas flooding for shorter durations (high *igapó*; Figure 1).

This predictable yet intense seasonal inundation drives profound physicochemical changes in soils, resulting in acidic, nutrient‐poor conditions that strongly constrain biological communities. As a consequence, the nutrient‐depleted waters of the Rio Negro basin support ecosystems characterized by relatively low overall species richness but exceptionally high endemism and species turnover (Junk et al. 1989, 2011, 2024; Kubitzki 1989; Ríos‐Villamizar et al. 2020; Wittmann et al. 2022; Householder et al. 2024). These environmental filters operate consistently across *igapó* systems, shaping community assembly along the flooding gradient.

Despite pronounced floristic turnover among *igapó* forests from different river systems, comparative studies indicate that they share remarkably similar structural organization across topographic levels. Distinct tree assemblages associated with high, medium, and low *igapós* have been documented in multiple regions (Ferreira 2000; Lobo et al. 2019), while herbaceous communities exhibit particularly high dissimilarity among sites (Lopes et al. 2014). At the basin scale, arboreal composition is dominated by a relatively small number of plant families, including Fabaceae, Lecythidaceae, Annonaceae, Sapotaceae, Myrtaceae, Moraceae, Chrysobalanaceae, and Lauraceae (Ferreira and Stohlgren 1999; Montero et al. 2012; Scudeller and Villarrúbia 2018), suggesting convergence in functional strategies despite taxonomic differences.

The persistence of trees in these environments is made possible by a suite of morphological, anatomical, and physiological adaptations that enable survival under prolonged inundation. These include the development of aerenchyma, adventitious roots, and mechanisms for rhizosphere oxidation, which facilitate oxygen transport to belowground tissues (Wittmann and Parolin 2005; Wittmann et al. 2010; Haase and Rätsch 2010). Consequently, the distribution of tree species along the flooding gradient is largely determined by their tolerance to inundation. These adaptations not only allow trees to survive flooding but also directly influence their interactions with soil biota, particularly microorganisms involved in nutrient acquisition.

Among the most important belowground interactions in *igapó* forests are mutualistic associations with mycorrhizal fungi. Mycorrhizae are one of the most widespread plant–microbe interactions (Rinaldi et al. 2008; Voller et al. 2023). The symbiosis enhances the absorption of water and nutrients by the plant partner (N, P, K, and Ca) in return for photosynthesis products, thereby increasing plant tolerance to drought, high temperatures, and soil acidity (Watkinson et al. 2016). Also, mycorrhizae facilitate seedling establishment and growth (Watkinson et al. 2016), potentially promoting the dominance of some tree species in nutrient‐poor tropical ecosystems like *igapós* by reducing pathogen‐related negative feedback (Alexopoulos et al. 1996; Smith et al. 2011; Roy et al. 2016). Additionally, mycorrhizal fungi fulfill important functions on the ecosystem level, for instance improving soil structure, fertility, and salinity (Courty et al. 2010; Wang and Rengel 2024). Most vascular plants form some type of mycorrhiza, which differs in the anatomical structure and taxonomic group of the fungal interaction partner: ~72% of plant species interact with arbuscular mycorrhizal fungi (AMF), 2% with ectomycorrhizal fungi (ECM), whereas 10% form orchid mycorrhizae, and 1.5% form ericoid mycorrhizae (Brundrett and Tedersoo 2018).

In Amazonian trees, both AMF and ECM associations occur (Vasco‐Palacios et al. 2020). AMF belong to the monophyletic group Glomeromycota and are characterized by hyphae that penetrate root epidermal cells and extend into the cortex. ECM, in contrast, do not penetrate root cells; their hyphae form a mycelial sheath (mantle) around the roots and spread through intercellular spaces forming a specific structure, the Hartig net, in the outer cortex, expanding contact with the substrate and offering protection from pathogens (Alexopoulos et al. 1996; Watkinson et al. 2016; Halling 2001; Lutzoni et al. 2018).

Early surveys of fungi in Amazonian *igapó* forests were largely based on sporome observations and inferred associations. Singer and Aguiar (1986) reported 49 fungal species in the Tarumã‐Mirim River, although later work demonstrated that many of these taxa were not ectomycorrhizal. Nevertheless, families such as Amanitaceae, Boletaceae, and Russulaceae have been consistently reported as ectomycorrhizal in Amazonian forests (Tedersoo and Brundrett 2017; Brundrett and Tedersoo 2018; Corrales et al. 2018). More recently, metabarcoding studies have transformed our understanding of fungal diversity. Ritter et al. (2020) showed that habitat type is the primary determinant of fungal community composition across Amazonian ecosystems, surpassing soil and locality effects. In flooded forests, particularly *várzea*, the widespread distribution of fungal ASVs along flooding gradients has been attributed to DNA transport by floodwaters, suggesting that relative abundance and community composition may be more informative than presence–absence patterns alone (Bredin et al. 2021).

As observed for tree communities, ectomycorrhizal fungi exhibit high spatial turnover in tropical forests. Strong compositional changes have been documented over short distances and along fertility gradients (Corrales et al. 2018), patterns comparable to those described for *igapó* forests (Furch 1997). Globally, ectomycorrhizal fungi associate with a limited subset of plant lineages but infect a substantial proportion of tree species, with their distribution shaped by climatic, spatial, and edaphic factors (Tedersoo et al. 2014; Tedersoo and Brundrett 2017; Steidinger et al. 2019). In the Amazon, local dominance of ectomycorrhizal host trees can promote high fungal diversity, as observed in monodominant or host‐rich systems such as *Dicymbe*‐dominated forests and white‐sand ecosystems (Henkel et al. 2002; Roy et al. 2016; Ritter et al. 2020).

Recent studies highlight the high turnover of mycorrhizal fungi across soil moisture and fertility gradients in tropical regions (Corrales et al. 2018; Vasco‐Palacios et al. 2020) and global studies suggest that ectomycorrhizal diversity tends to be greater in forests dominated by one or a few tree species (Voller et al. 2023). This pattern contrasts with early work in Amazonian *igapós*, where higher ECM richness was reported in high *igapós* despite their greater tree diversity, while low *igapós*, with fewer tree species and more prolonged flooding, appeared to support lower ECM richness (Singer and Aguiar 1986). These results stress the Amazonian *igapós* as an exciting, but understudied, system to understand how flood duration and severity shape mycorrhizal communities. In particular, the local mycorrhizal fungi richness may peak in high *igapós* and decrease linearly toward low *igapó* as only more and more specialized species are able to cope with the prolonged flooding (Cho et al. 2021), or it may peak at intermediate disturbance combining species tolerant of both extremes (Medium *igapós*; Dial and Roughgarden 1998; Zhang et al. 2018). Furthermore, it is unclear whether ectomycorrhizal fungi partition niches across *igapó* topographies (Singer and Aguiar 1986). This knowledge gap complicates predictions of *igapó* tree responses to changes in the drought‐inundation cycle. The challenge partly stems from the difficulty of identifying mycorrhizal taxa using anatomical methods. In this context, DNA metabarcoding offers a more accurate alternative, particularly useful for remote areas (Bredin et al. 2021; Ritter et al. 2020). This technique enables precise fungal community analyses, detecting rare species and tracking ecosystem changes over time (Tedersoo, May, and Smith 2010; Tedersoo, Sadam, et al. 2010; Tedersoo et al. 2014, 2017, 2024; Tedersoo, Bahram, et al. 2022; Tedersoo, Mikryukov, et al. 2022; Corrales et al. 2018; Semenov 2021).

Here, we used DNA metabarcoding of root samples from three Amazonian *igapó* forests to characterize mycorrhizal fungal richness and community composition and to evaluate the influence of flood regimes using topographic position as a proxy, while explicitly accounting for geographic site effects. We hypothesized that (H1) mycorrhizal richness and diversity, as well as the relative contribution of ectomycorrhizal fungi, are higher in medium and high *igapós* than in low *igapós*, where flooding is more intense and tree communities are more strongly filtered; and (H2) mycorrhizal community composition differs among sites and among *igapó* topographic levels, reflecting spatial structuring driven by geographic context and flooding‐related environmental gradients in the Central Amazonia.

## 2 Material and Methods

### 2.1 Study Area and Sampling Design

To verify the effect of flooding on tree‐mycorrhizal fungus interactions, we selected three *igapó* sites in the Negro River basin, with a well‐documented flora (Figure 1; PELD—MAUA/INPA database—Long‐term Monitoring Program for Wetlands). At each site, we sampled roots in rectangular plots (Table S1), where all trees with diameter at breast height (DBH) ≥ 10 cm were identified and mapped. The first area was RDS Uatumã (Uatumã Sustainable Development Reserve) at the Abacate River, located on the border between the municipalities of Presidente Figueiredo and São Sebastião do Uatumã, Amazonas state (AM), Brazil. The second area was the RDS Rio Negro (Rio Negro Sustainable Development Reserve) at the Cuieiras River, situated near Manaus, AM, Brazil. The third area was the PARNA Jaú (Jaú National Park) at the Jaú River, located in the municipality of Novo Airão, AM, Brazil. The Jaú River drains 10,000 km^2^ of tropical forest and meets the main channel of the Rio Negro about 300 km upstream from its mouth. Its smaller tributaries are flooded annually, with varying frequency and duration (Forsberg et al. 2001; Junk et al. 2011, 2014). The Uatumã floodplain is wide and experiences strong seasonal pulses (Walker et al. 1999; Kasper et al. 2014). The Cuieiras River, similar to the Uatumã, is also heavily impacted by the flood pulse.

For root sampling, we targeted host trees selected through a stratified approach to ensure a representative yet biologically meaningful collection of tree species following three criteria: (1) abundance, including dominant and rare tree species within each *igapó* locality and topographic level, as quantified by field surveys and the long‐term PELD‐MAUA forest inventory (Table 1); (2) taxonomic predisposition as ECM host, prioritizing species from genera or families with documented ectomycorrhizal associations in the literature; and (3) direct fungal evidence, focusing on trees in immediate proximity to basidiomes of known ectomycorrhizal taxa (e.g., *Russula*, *Entoloma*, *Amanita*, boletes genera). We followed this tripartite strategy to maximize the probability of finding mycorrhizal fungi and to address the inherent ecological variability of *igapó* forests, where flood‐mediated turnover in tree community composition creates heterogeneous host availability across sites. The inclusion of rare species alongside dominant taxa increased the potential to capture niche‐specific partnerships, while the basidiome proximity criterion allowed field‐verifiable confirmation of active symbioses, minimizing false negatives in root sampling. Variable sampling effort per species arose from this framework: host abundance differed among *igapós*, and ectomycorrhizal associations were detectable only where fungal fruiting bodies occurred or records already exist in literature. Consequently, sample sizes reflect host distribution and symbiosis frequency rather than a balanced experimental design. This approach aligns with the study's goal of characterizing ectomycorrhizal networks under natural conditions, where flood‐driven disturbances and host specificity jointly shape fungal colonization patterns.

**TABLE 1:** Sampling sites and corresponding flood regime.

Locality	Plot	Topography	Flooding level± (m)
RDS UATUMÃ—Abacate River	A1A	Low	≥ 5
RDS UATUMÃ—Abacate River	A1B	Low	≥ 5
RDS UATUMÃ—Abacate River	A2A	Medium	3–4
RDS UATUMÃ—Abacate River	A2B	Medium	3–4
RDS UATUMÃ—Abacate River	A3A	High	1–2
RDS RIO NEGRO—Cuieiras River	CA1	High	1–2
RDS RIO NEGRO— Cuieiras River	CA2	Medium	3–4
RDS RIO NEGRO—Cuieiras River	CA3	Low	≥ 5
PARNA JAU—Jaú River	JP1	Low	≥ 5
PARNA JAU—Jaú River	JP5	Medium	3–4
PARNA JAU—Jaú River	JP6	Medium	3–4
PARNA JAU—Jaú River	JP7	High	1–2
PARNA JAU—Jaú River	JP9	High	1–2

We collected fine root samples (30 mg) from 157 tree individuals belonging to 68 species (Table 2) between August and December (the dry season when *igapós* were drained) from 2019 to 2021. We identified tree species based on morphological characteristics using identification keys in the field and based on material deposited in the Herbarium of the National Institute of Amazonian Research (INPA). We took the root samples by removing individual roots from the plant's root systems close to the soil surface, and subsequently packaged them in paper envelopes. In the laboratory, the samples were cleaned and packaged in zipped bags with silica gel and kept in a freezer at an average temperature of −15°C. All sampling was done with legal permission (SISBIO license 66334‐1).

**TABLE 2:** Summary of sampling effort and metabarcoding output by site and plot.

Site	Plot	Topography	Plant species	Individuals sampled (*n*)	Reads (all mycorrhizae)	ASVs (all mycorrhizae)	Reads (AMF)	ASVs (AMF)	Reads (ECM)	ASVs (ECM)
PARNA JAU	JP1	Low	*Acosmium nitens*	1	170	19	157	13	13	6
PARNA JAU	JP1	Low	*Amanoa oblongifolia*	2	309	36	181	16	127	19
PARNA JAU	JP1	Low	*Eschweilera tenuifolia*	2	5203	42	209	26	371	14
PARNA JAU	JP1	Low	*Ormosia excelsa*	1	18	7	9	5	5	1
PARNA JAU	JP5	Mid	*Aldina latifolia*	1	524	19	63	11	461	8
PARNA JAU	JP5	Mid	*Elvasia* sp.	1	261	31	216	24	45	7
PARNA JAU	JP5	Mid	*Erythroxylum spruceanum*	1	247	10	168	8	0	0
PARNA JAU	JP5	Mid	*Pouteria elegans*	2	2915	37	286	17	2629	20
PARNA JAU	JP5	Mid	*Swartzia laevicarpa*	1	414	23	119	13	295	10
PARNA JAU	JP5	Mid	*Swartzia polyphylla*	1	381	28	9	3	372	25
PARNA JAU	JP5	Mid	*Tachigali* sp.	2	1792	65	706	27	1065	36
PARNA JAU	JP5	Mid	*Terminalia* sp.	1	672	17	403	6	269	11
PARNA JAU	JP5	Mid	*Tovomita spruceana*	1	88	5	0	0	88	5
PARNA JAU	JP6	Mid	*Amanoa oblongifolia*	2	269	33	172	21	35	9
PARNA JAU	JP6	Mid	*Burdachia* sp.	2	896	30	613	7	283	23
PARNA JAU	JP6	Mid	*Duroia velutina*	1	105	16	1	1	104	15
PARNA JAU	JP6	Mid	*Hydrochorea marginata*	1	13	5	4	2	9	3
PARNA JAU	JP6	Mid	*Maprounea amazonica*	1	8011	20	828	11	7183	9
PARNA JAU	JP6	Mid	*Micropholis humboldtiana*	1	782	32	205	16	557	13
PARNA JAU	JP7	High	*Alchorneopsis floribunda*	1	138	27	79	12	55	14
PARNA JAU	JP7	High	*Aldina latifolia*	3	14,821	83	385	20	14,043	59
PARNA JAU	JP7	High	*Campsiandra angustifolia*	1	4084	63	5	2	4079	61
PARNA JAU	JP7	High	*Dialium guianense*	1	11,061	42	335	17	10,653	23
PARNA JAU	JP7	High	*Eschweilera* sp.	2	17,177	42	128	4	17,049	38
PARNA JAU	JP7	High	*Eschweilera* sp.*3*	1	94	10	9	4	85	6
PARNA JAU	JP7	High	*Macrolobium acaciifolium*	1	452	29	2	2	450	27
PARNA JAU	JP7	High	*Miconia poeppigii*	1	5442	41	63	7	5374	32
PARNA JAU	JP7	High	*Swartzia laevicarpa*	1	845	40	193	18	181	17
PARNA JAU	JP7	High	*Vatairea guianensis*	1	420	23	0	0	420	23
PARNA JAU	JP9	High	*Aldina latifolia*	1	854	28	243	15	611	13
PARNA JAU	JP9	High	*Brosimum guianense*	1	12	8	8	6	4	2
PARNA JAU	JP9	High	*Elvasia quinqueloba*	2	668	42	293	33	375	9
PARNA JAU	JP9	High	*Licania adolphoduckei*	1	190	29	123	23	67	6
PARNA JAU	JP9	High	*Mouriri* sp.	1	81	16	41	12	40	4
PARNA JAU	JP9	High	*Pouteria elegans*	1	480	24	202	12	278	12
PARNA JAU	JP9	High	*Sacoglottis guianensis*	1	516	7	18	3	498	4
PARNA JAU	JP9	High	*Tachigali* sp.	2	1346	36	747	21	599	15
RDS RIONEGRO	CA1	High	*Abarema jupunba*	1	219	12	73	9	146	3
RDS RIONEGRO	CA1	High	*Acosmium nitens*	4	5843	64	2450	49	3380	14
RDS RIONEGRO	CA1	High	*Aspidosperma pachypterum*	1	1284	12	870	6	413	5
RDS RIONEGRO	CA1	High	*Cybianthus densiflorus*	1	1240	15	771	7	469	8
RDS RIONEGRO	CA1	High	*Elvasia calophyllea*	1	2071	21	1514	14	557	7
RDS RIONEGRO	CA1	High	*Licania apetala*	1	890	28	678	21	212	7
RDS RIONEGRO	CA1	High	*Micropholis humboldtiana*	2	1328	19	564	16	764	3
RDS RIONEGRO	CA1	High	*Mouriri* sp.	2	2712	39	1560	23	1073	15
RDS RIONEGRO	CA1	High	*Pouteria elegans*	5	7674	69	4172	45	3502	24
RDS RIONEGRO	CA1	High	*Swartzia acuminata*	2	2931	26	385	10	2546	16
RDS RIONEGRO	CA1	High	*Tachigali* sp.	1	141	2	141	2	0	0
RDS RIONEGRO	CA1	High	*Tovomita* sp.	2	1613	19	780	14	833	5
RDS RIONEGRO	CA2	Mid	*Calypthranthes* sp.	1	914	27	697	20	207	6
RDS RIONEGRO	CA2	Mid	*Micropholis humboldtiana*	1	1061	23	1035	20	26	3
RDS RIONEGRO	CA2	Mid	*Pouteria elegans*	2	4023	27	3960	23	63	4
RDS RIONEGRO	CA2	Mid	*Swartzia acuminata*	1	7766	47	441	15	7307	31
RDS RIONEGRO	CA2	Mid	*Tachigali oppositifolia*	1	1241	43	682	26	559	17
RDS RIONEGRO	CA3	Low	*Calypthranthes* sp.	1	4955	45	623	16	1848	23
RDS RIONEGRO	CA3	Low	*Duroia velutina*	1	1327	22	213	9	285	10
RDS RIONEGRO	CA3	Low	*Licania* sp.	1	587	13	37	6	43	4
RDS RIONEGRO	CA3	Low	*Micropholis humboldtiana*	2	10,464	49	4573	34	439	13
RDS RIONEGRO	CA3	Low	*Swartzia polyphylla*	1	424	19	281	14	143	5
RDS RIONEGRO	CA3	Low	*Swartzia* sp.	1	258	10	43	4	10	4
RDS UATUMA	A1A	Low	*Campsiandra comosa*	2	12,236	65	1190	46	11,043	18
RDS UATUMA	A1A	Low	*Caraipa richardiana*	2	776	24	564	14	164	9
RDS UATUMA	A1A	Low	*Couratari tenuicarpa*	2	497	19	86	5	410	13
RDS UATUMA	A1A	Low	*Crudia amazonica*	2	1161	56	813	42	347	13
RDS UATUMA	A1A	Low	*Eperua duckeana*	1	1413	42	807	11	606	31
RDS UATUMA	A1A	Low	*Eschweilera albiflora*	3	3577	83	1564	51	963	27
RDS UATUMA	A1A	Low	*Eugenia lambertiana*	3	2941	113	2285	86	622	24
RDS UATUMA	A1A	Low	*Macrolobium angustifolium*	1	292	9	104	5	188	4
RDS UATUMA	A1A	Low	*Mouriri brevipes*	1	2479	46	1419	41	1035	4
RDS UATUMA	A1A	Low	*Swartzia laevicarpa*	1	338	49	236	38	101	10
RDS UATUMA	A1A	Low	*Swartzia polyphylla*	2	675	49	343	32	323	15
RDS UATUMA	A1A	Low	*Zygia cataractae*	4	5657	117	581	46	5076	71
RDS UATUMA	A1B	Low	*Amanoa oblongifolia*	1	11,850	28	550	22	11,300	6
RDS UATUMA	A1B	Low	*Calophyllum brasiliense*	1	667	30	378	15	288	14
RDS UATUMA	A1B	Low	*Campsiandra comosa*	2	708	59	131	16	577	43
RDS UATUMA	A1B	Low	*Caraipa richardiana*	4	2187	47	457	22	1361	22
RDS UATUMA	A1B	Low	*Couratari cf. tenuicarpa*	1	531	37	156	22	147	12
RDS UATUMA	A1B	Low	*Couratari tenuicarpa*	1	477	30	459	28	18	2
RDS UATUMA	A1B	Low	*Crudia amazonica*	3	1937	73	990	43	885	28
RDS UATUMA	A1B	Low	*Dicorynia paraensis*	1	2383	45	2215	27	136	17
RDS UATUMA	A1B	Low	*Elvasia calophyllea*	1	1240	41	357	28	75	11
RDS UATUMA	A1B	Low	*Eschweilera albiflora*	5	20,137	102	1515	68	18,431	33
RDS UATUMA	A1B	Low	*Eschweilera cf. albiflora*	1	424	39	277	24	128	12
RDS UATUMA	A1B	Low	*Manilkara bidentata*	2	1523	66	630	27	869	38
RDS UATUMA	A1B	Low	*Micropholis melinoniana*	1	404	29	343	27	1	1
RDS UATUMA	A1B	Low	*Ocotea aciphylla*	1	405	33	194	15	211	18
RDS UATUMA	A1B	Low	*Sacoglottis guianensis*	2	855	39	129	21	577	15
RDS UATUMA	A1B	Low	*Swartzia laevicarpa*	1	59,404	35	60	21	59,312	12
RDS UATUMA	A1B	Low	*Unonopsis guatterioides*	1	13	9	10	7	3	2
RDS UATUMA	A1B	Low	*Zygia cataractae*	3	487	57	60	14	426	42
RDS UATUMA	A2A	Mid	*Aspidosperma exelsum*	1	497	22	309	17	152	4
RDS UATUMA	A2A	Mid	*Caraipa richardiana*	1	108	6	26	2	82	4
RDS UATUMA	A2A	Mid	*Eperua rubiginosa*	1	59	18	0	0	59	18
RDS UATUMA	A2A	Mid	*Humiriastrum cuspidatum*	1	6394	49	1261	36	4294	8
RDS UATUMA	A2A	Mid	*Licania hypoleuca*	1	518	19	451	13	67	6
RDS UATUMA	A2B	Mid	*Blepharocalyx eggersii*	1	413	12	14	3	20	5
RDS UATUMA	A2B	Mid	*Campsiandra comosa*	1	160	14	45	9	115	5
RDS UATUMA	A2B	Mid	*Licaria cannella*	1	1047	61	549	31	498	30
RDS UATUMA	A3A	High	*Campsiandra comosa*	2	11,920	63	2266	38	2079	17
RDS UATUMA	A3A	High	*Eschweilera albiflora*	3	2851	45	542	28	322	11
RDS UATUMA	A3A	High	*Eschweilera tessmannii*	1	2123	30	769	15	1352	14
RDS UATUMA	A3A	High	*Licania apetala*	1	457	16	146	8	250	7
RDS UATUMA	A3A	High	*Unonopsis guatterioides*	1	1105	22	335	14	770	8
RDS UATUMA	A3A	High	*Zygia cataractae*	1	62	11	9	6	53	5

*Note:* For each site × plot combination, we report the number of individuals for each tree species, total sequencing reads, and the number of detected ASVs for all mycorrhizae, arboreal mycorrhizae (AMF), and ectomycorrhizae (ECM).

### 2.2 DNA Isolation and Quantification

Under the SisGen number AAB7D27, samples were sent to the AllGenetics & Biology SL (www.allgenetics.eu; Spain) for DNA extraction, amplification, and sequencing. Meta DNA was extracted from each sample using the DNeasy PowerSoil Pro kit (QIAGEN). Up to 30 mg of root tips tissue from each sample, cut into small pieces with flame‐sterilized scissors and tweezers, was placed in the PowerBead Pro tubes provided with the kit, along with a lysis buffer. The samples were then grounded in the TissueLyser LT (QIAGEN) at 50 Hz for 5 min. DNA extraction precisely followed the manufacturer's instructions. An extraction blank (Bex1 to Bex7) was included in each round to monitor cross‐contamination, and these blanks were processed like regular samples. DNA was resuspended in a final volume of 50 μL. DNA concentrations were quantified using the Qubit High Sensitivity dsDNA Assay (Thermo Fisher Scientific).

### 2.3 DNA Metabarcoding Amplification and Sequencing

To identify fungi in the roots, a 300 bp fragment of the ITS2 region was targeted for amplification using the forward primer ITS86F (5′ GTGAATCATCGAATCTTTGAA 3′) (Turenne et al. 1999) and the reverse primer ITS4R (5′ TCCTCCGCTTATTGATATGC 3′) (White et al. 1990). This ITS2 fragment is widely used for fungal identification at the species level (White et al. 1990; Schoch et al. 2012; Kõljalg et al. 2013). Primer sequences for Illumina sequencing were attached to the 5′ ends of these primers. Amplification was carried out in a 12.5 μL reaction volume, containing 1.25 μL of DNA template, 0.5 μM of each primer, 3.13 μL of Supreme NZYTaq 2× Green Master Mix (NZYTech), and ultrapure water up to 12.5 μL. The PCR conditions included an initial denaturation at 95°C for 5 min, followed by 30 cycles of 95°C for 30 s, 47°C for 45 s, 72°C for 45 s, and a final extension at 72°C for 7 min.

Oligonucleotide indices required for multiplexing libraries were attached in a second amplification step under identical conditions but with only 5 cycles and a 60°C annealing temperature. A negative control without DNA (BPCR) was included in each PCR round to check for contamination. Library size was verified using 2% agarose gels stained with GreenSafe (NZYTech) and visualized under UV light. Libraries were purified using Mag‐Bind RXNPure Plus magnetic beads (Omega Biotek), following the manufacturer's instructions. Final libraries were pooled in equimolar amounts based on Qubit dsDNA HS Assay (Thermo Fisher Scientific) quantification and sequenced on a NovaSeq PE250 flow cell (Illumina), targeting a total output of 9 gigabases.

### 2.4 Sequence Analyses and Taxonomic Assessment

We produced amplicon sequence variants (ASVs) using the DADA2 v.1.16 package (Callahan et al. 2016) in R v. 4.3.0 (R Core Team 2024) to process the raw data. This included primer removal, quality filtering, sequence merging, chimera removal, ASV inference, and taxonomic assignment. Reads containing ambiguous bases (maxN = 0) were excluded. Quality filtering was based on the error rates of the forward and reverse sequences, allowing a maximum of three errors for forward reads and five errors for reverse reads (maxEE = c(3,5), truncQ = 2). ASVs were inferred using run‐specific error rates for each sample. Paired‐end reads were merged with a minimum overlap of 12 base pairs, and sequences with mismatches in the overlapping region were discarded. Taxonomic assignment of ASVs was performed using the UNITE database v.16.10.2022 (Abarenkov et al. 2010). Additionally, functional guilds were assigned to ASVs identified as fungi using the FungalTraits database v.0.0.3 (Põlme et al. 2020), with manual verification of the assignments to ensure accuracy.

### 2.5 Statistical Analysis

We conducted all analyses in the R environment. We used the tidyverse package v.1.3.0 (Wickham et al. 2019) for data curation and ggplot2 v. 3.3.2 (Wickham 2016), ggfortify v. 0.4.11 (Tang et al. 2016), and gridExtra v. 2.3 (Auguie and Antonov 2017), for data visualization (scripts in Appendix S1).

For all analyses, we only retained ASV identified as belonging to mycorrhizal fungi in the FungalTraits database. We calculated the ASV richness (the number of ASV per tree), Shannon's index, and inverse Simpson's index for alpha diversity using the phyloseq v.1.34.0 (McMurdie and Holmes 2013) R package. To evaluate whether mycorrhizal alpha diversity varied across localities and flood levels, we applied Kruskal–Wallis test, followed by post hoc Dunn tests with Bonferroni correction for pairwise comparisons using the dunn.test V.1.3.6 R package (Dinno and Dinno 2017). All analyses were conducted separately for the total observed mycorrhizal fungi and for the subset of ECM.

We explored beta diversity patterns using non‐metric multidimensional scaling (NMDS) ordinations. NMDS was performed both on relative abundance data using Bray–Curtis distances and on presence/absence data using Jaccard distances with the phyloseq package. To test the significance of the difference in community composition for flood level and localities, we applied PERMANOVA with 999 permutations on both Bray–Curtis and Jaccard distances for relative abundance and presence and absence matrix, respectively, with the vegan v.2.6.10 R package (Oksanen et al. 2013). Finally, we tested for homogeneity of multivariate dispersions across groups using the betadisper function of the vegan package, ensuring that PERMANOVA results could be interpreted in terms of compositional differences rather than unequal dispersions.

## 3 Results

We found a total of 11,043 ASVs of which 7760 were assigned as fungi. Of those, we identified 568 as mycorrhizal fungi: 238 as arbuscular mycorrhizal fungi (from the division Glomeromycota; AMF), 307 as ectomycorrhizal fungi from various taxa (ECM), three as ericoid mycorrhizal fungi, and 20 as mycorrhizal fungi of unidentified type and taxon. The Number of ASVs per tree is available in Table 2.

**FIGURE 2:**
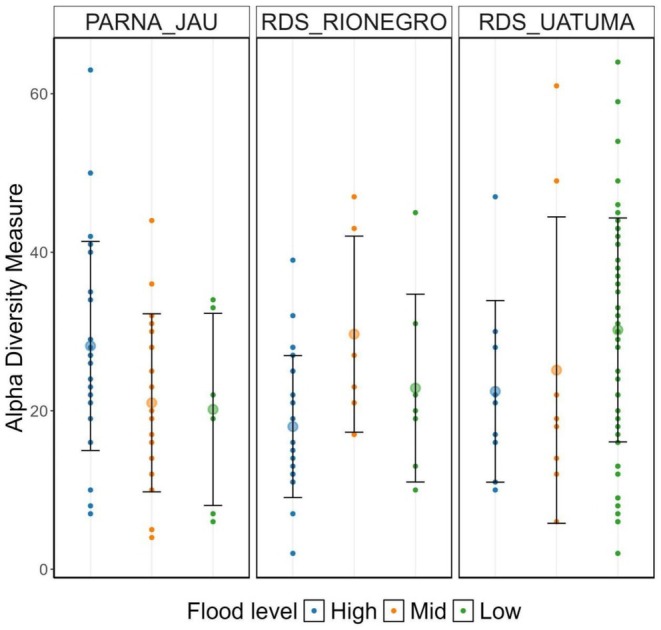
Alpha diversity of mycorrhizal amplicon sequence variants (ASVs), including arbuscular (AMF), ectomycorrhizal (ECM), ericoid, and unidentified mycorrhizal fungi, from roots across flood levels (High, Mid, Low) in three Amazonian *igapó* sites: PARNA Jaú, RDS Rio Negro, and RDS Uatumã. Each point represents the number of different mycorrhizal ASVs observed per sample plot. Larger circles indicate the mean and the bars represent the standard deviation per flood level within each site. For Shannon and inverse Simpson diversity indexes, figures are available at Supporting Information (Figures S1 and S2).

Across flood levels, the total mycorrhizal fungi ASV richness per sample varied significantly (Kruskal–Wallis test: *χ* ^2^ = 7.36, *p* = 0.02; Figure 2). The post hoc Dunn tests revealed that the total mycorrhizal fungi ASV richness was significantly higher in low versus high *igapós* (*p* = 0.02), but no significant difference between low versus medium (*p* = 0.07) and medium versus high *igapós* (*p* = 1; Figure 2, Figures S1 and S2). In contrast, neither the inverse Simpson diversity (Kruskal–Wallis test: *χ* ^2^ = 3.14, *p* = 0.2) nor the Shannon diversity (Kruskal–Wallis test: *χ* ^2^ = 5.23, *p* = 0.07) varied significantly across flood levels for total mycorrhizal fungi ASVs.

Across localities, the total mycorrhizal fungi ASVs richness also varied, with 416 ASVs recorded in RDS Uatumã, 221 in RDS Rio Negro, and 321 in PARNA Jaú. This difference was significant (*χ* ^2^ = 8.42, *p* = 0.01; Figure 2). The post hoc Dunn tests indicated that RDS Uatumã had significantly higher total mycorrhizal richness than RDS Rio Negro (*p* = 0.007), while no significant differences were detected between PARNA Jaú and the other sites. The Shannon (Kruskal–Wallis test: *χ* ^2^ = 8.91, *p* = 0.01, Figure 2) and inverse Simpson (Kruskal–Wallis test: *χ* ^2^ = 6.36, *p* = 0.04) diversity also indicated a significant difference.

**FIGURE 3:**
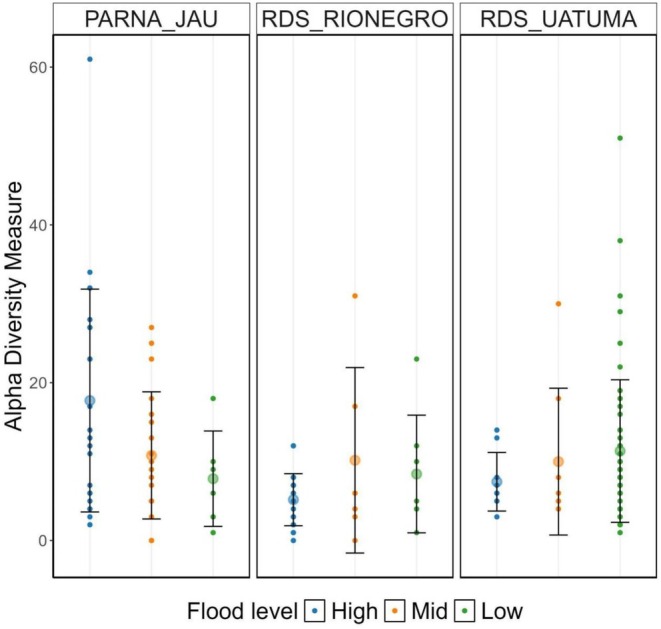
Alpha diversity of ectomycorrhizal ASVs from roots across flood levels (High, Mid, Low) in three Amazonian *igapó* sites: PARNA Jaú, RDS Rio Negro, and RDS Uatumã. Each point represents the richness of mycorrhizal amplicon sequence variants (ASVs) observed per plot. Larger circles and bars indicate the mean, and the bars represent the standard deviation per flood level within each site.

Focussing on the ECM, ASVs richness did not vary significantly across flood levels (Kruskal–Wallis test: *χ* ^2^ = 1.28, *p* = 0.52, Figure 3) but varied significantly across location, with 416 ASVs recorded in RDS Uatumã, 224 in RDS Rio Negro, and 321 in PARNA Jaú (Kruskal–Wallis test: *χ* ^2^ = 14.5, *p* < 0.001; Figure 3). The Dunn test showed that RDS Rio Negro is significantly different from PARNA Jaú (*p* < 0.001) and RDS Uatumã (*p* = 0.003).

**FIGURE 4:**
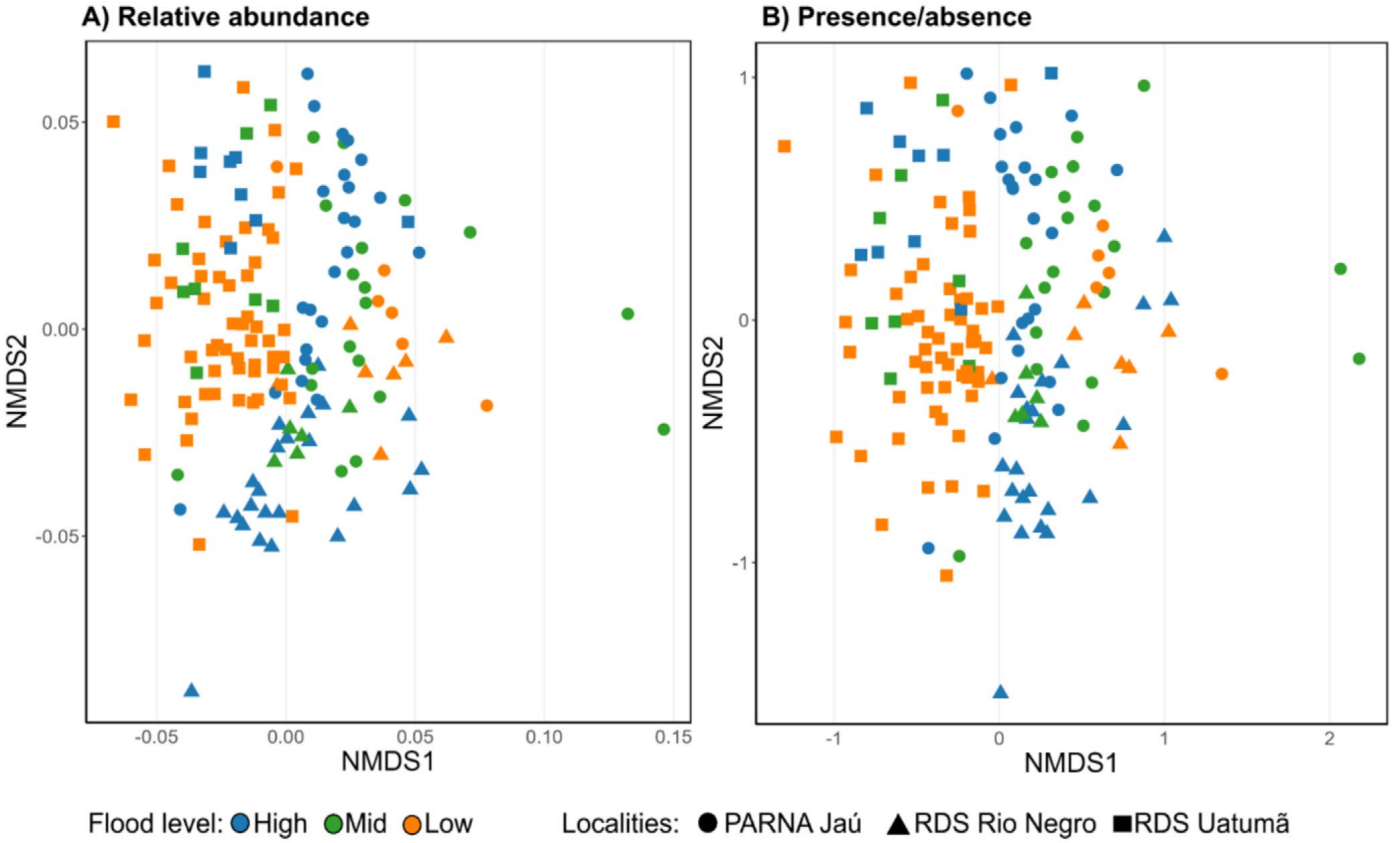
Non‐metric multidimensional scaling (NMDS) of mycorrhizal ASV communities across flood levels and localities. (A) Relative‐abundance (Bray–Curtis; stress ≈0.25) and (B) presence/absence (Jaccard; stress ≈0.23) ordinations. Colors indicate flood level (High, Mid, Low) and shapes indicate locality (circle = PARNA Jaú; triangle = RDS Rio Negro; square = RDS Uatumã). Ordinations show broad overlap among groups with subtle shifts along NMDS1/NMDS2.

Concerning community composition, the non‐metric multidimensional scaling (NMDS) based on Bray–Curtis and Jaccard dissimilarities showed a substantial overlap among samples from different localities and flood levels (Figure 4). Consistently, the PERMANOVA results indicate only small (albeit significant) effects of both topography and locality on community composition for relative abundance (Flood level: *R* ^2^ = 0.025, *F* = 2.08, *p* = 0.001; Area: *R* ^2^ = 0.050, *F* = 4.13, *p* = 0.001) and for presence/absence (Flood level: *R* ^2^ = 0.022, *F* = 1.82, *p* = 0.001; Area: *R* ^2^ = 0.052, *F* = 4.35, *p* = 0.001). The tests for homogeneity of dispersions indicated no significant differences across topographies (relative abundance: *p* = 0.87; presence/absence: *p* = 0.45), but significant differences across localities (relative abundance: *p* < 0.001; presence/absence: *p* = 0.06). These results suggest that the PERMANOVA effects for locality should be interpreted with caution, as they may be partly driven by differences in multivariate dispersion.

**FIGURE 5:**
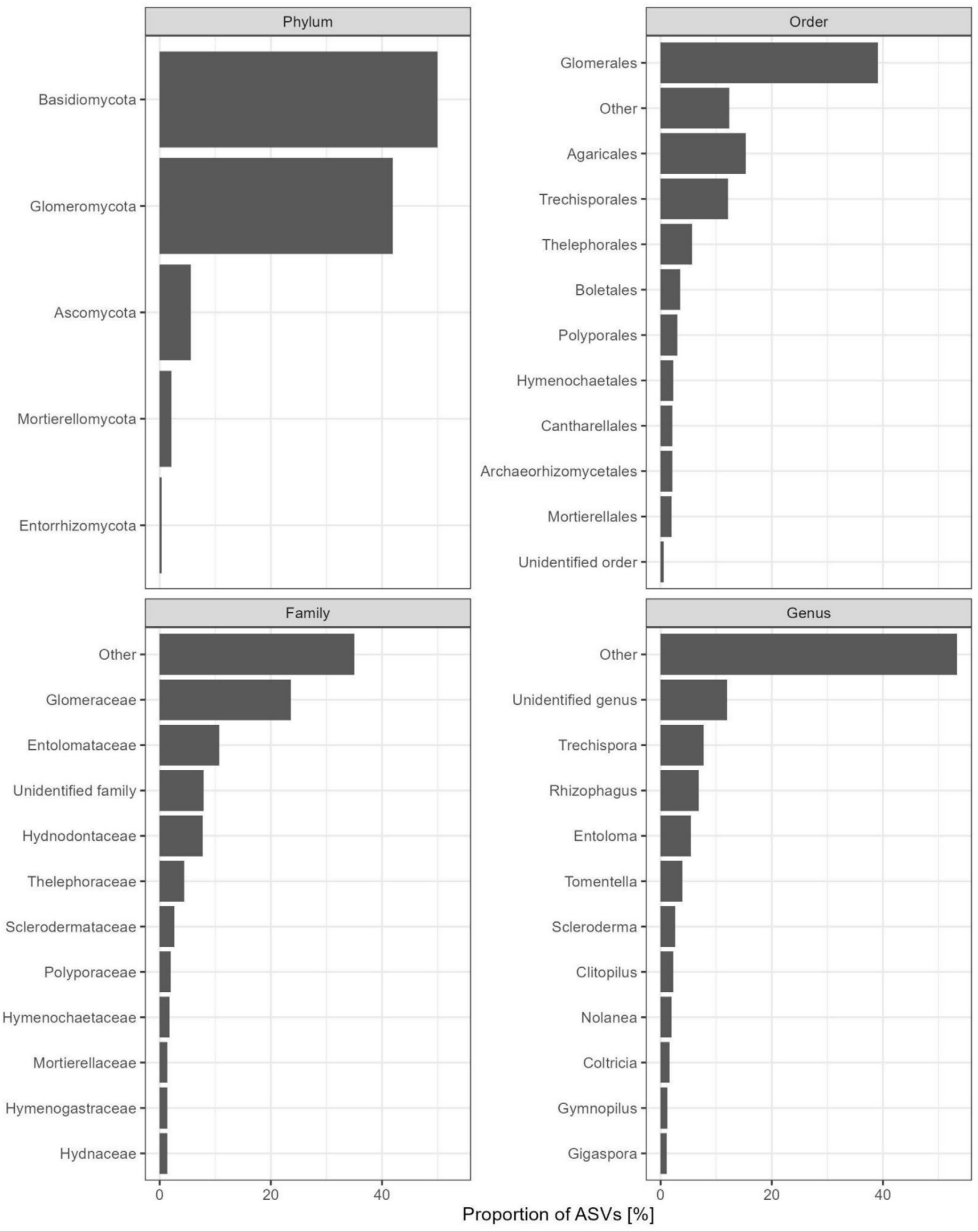
Proportion of mycorrhizal amplicon sequence variants (ASVs) assigned to major fungal taxa at different taxonomic ranks (phylum, order, family, genus), pooled across all root samples from the three *igapó* sites. The 10 most ASV‐rich taxa at each rank are named. Taxa with fewer ASVs are grouped as “Other,” and ASVs that could not be assigned at a given taxonomic rank are shown as separate “Unidentified” categories.

The presence and relative abundance of different taxonomic groups varied between localities (Figure 5). In PARNA Jaú, Archaeorhizomycetes, Entolomataceae (ECM), and Glomeraceae (AMF) were the dominant taxa, with other ECM families such as Hymenogastraceae, Russulaceae, Sclerodermataceae, and Thelephoraceae present at lower abundances. A similar taxonomic composition was observed in RDS Rio Negro, although relative abundances varied. Conversely, RDS Uatumã showed a distinct pattern, with a higher abundance of unidentified fungal lineages and a reversal in ECM‐AMF dominance: Entolomataceae (ECM) were more abundant than Glomeraceae (AMF). For taxonomic groups, for site and topography see Appendix Figures S1–S3.

## 4 Discussion

Our study revealed a significant variability in the alpha and beta diversity of mycorrhizal fungi across different *igapó* sites and flood levels. In contrast to our expectations (H1), mycorrhizal fungi ASV richness was highest in low‐level *igapós*, which experience longer flooding and are dominated by few tree species. However, this pattern was dependent on functional group and locality: for the ECM, richness was highest in high and mid‐level *igapós* at PARNA Jaú and RDS Rio Negro, whereas at RDS Uatumã the diversity was highest in low‐level *igapós*. It is important to note that richness metrics tend to overemphasize rare taxa; accordingly, for topographic effects, Shannon and inverse Simpson indices were not significant. These indices down‐weight rare taxa and are less sensitive to uneven sampling and in metabarcoding contexts with uneven sequencing depth (Tedersoo, Bahram, et al. 2022; Tedersoo, Mikryukov, et al. 2022). Taken together, our results suggest that flood‐level differences may occur primarily in the rare tail of the mycorrhizal community in certain contexts, while overall dominance structure and evenness were not consistently altered across flood levels when considering the full mycorrhizal assemblage. Mycorrhizal communities were modestly structured by flood‐level topography and locality, stressing that despite a superficial physiognomic homogeneity, central Amazonian *igapós* are diverse in mycorrhizal fungi composition at different sites (Householder et al. 2024). Correspondingly, the occurrence of fungal families and orders varied across topographic levels, reflecting the influence of flooding and host tree species.

### 4.1 Alpha Diversity

The data presented in this study enrich existing information on the mycorrhizal communities of Amazonia: a recent review of global fungi data had revealed that all of the 1362 available metabarcoding samples from the Neotropics combined only documented 406 ECM fungal lineages (Corrales et al. 2022). In contrast, our study of the central Amazonian *igapós*, a previously undersampled ecosystem, alone identified 307 unique ASVs of ECM fungi, underscoring the remarkable diversity, potential endemism, and need for further research of these communities (Tedersoo et al. 2014, 2024).

The documented richness patterns of overall mycorrhizal fungi (including AMF, ECM, and ericoid mycorrhiza) among the studied rivers are striking. The study trees at PARNA Jaú emerge as a hotspot for mycorrhizal diversity, particularly in high‐level *igapós* where all mycorrhizal types co‐occur at their highest richness. This pattern contrasts sharply with the Abacate River (RDS Uatumã) system, where maximum diversity occurs in low‐lying, frequently flooded areas. The Cuieiras River (RDS Rio Negro) presents an intermediate scenario, with peak diversity concentrated in mid‐level *igapós*. Notably, Glomeraceae (AMF) reaches its highest abundance in the Cuieiras River and the Jaú River. Intermediate flood levels fall between these two extremes. The reasons for these differences among rivers need verification on a larger geographic scale with a larger sample size from a comparative sampling scheme, but may be related to river structure and origin. The Jaú drains weathered, nutrient‐poor soils, with high concentrations of dissolved organic carbon derived from hydromorphic soils (Forsberg et al. 2001). The Uatumã River flows through the Guiana Shield and the central Amazon sedimentary basin, where clayey and sandy soils predominate (Ríos‐Villamizar et al. 2020).

### 4.2 Beta Diversity

Our study reveals that mycorrhizal fungal assemblages in Amazonian *igapó* forests are structured primarily by locality, with flood‐level topography acting as a secondary, context‐dependent filter. Although overall community overlap was high across samples, multivariate analyses consistently detected significant effects of both basin and flood level on community composition. These effects were modest in magnitude, yet robust across abundance‐weighted (Bray–Curtis) and occurrence‐based (Jaccard) dissimilarities, indicating that differences among sites are expressed both in shifts in dominant lineages and in the distribution of rarer taxa.

The relatively small effect sizes observed for both locality and topography are ecologically expected in *igapó* systems, which share broad physiognomic similarity but differ subtly in geomorphology, hydrological regime, and floristic composition (Montero et al. 2012; Montero and Latrubesse 2013; Scudeller and Villarrúbia 2018). Tests of multivariate dispersion further indicate that locality effects may partly reflect differences in within‐site heterogeneity, suggesting that basins differ not only in the central tendency of their fungal communities but also in the breadth of assemblages that can establish locally. Taken together, these results support a view of *igapó* forests as a mosaic of belowground communities shaped by basin‐scale environmental context and filtered by flood‐related gradients.

### 4.3 Taxonomic Composition

Taxonomic summaries across phylum, order, family, and genus levels provide biologically meaningful context for these compositional patterns. Across all sites and flood levels, mycorrhizal communities shared a consistent high‐level structure dominated by Basidiomycota (including ECM and orchid mycorrhizae taxa), Glomeromycota (AMF), and Ascomycota (ECM and ericoid mycorrhizae), reflecting the widespread presence of ECM and AMF fungi in *igapó* root systems. This stability at higher taxonomic ranks contrasts with clearer differentiation at finer levels, where basin‐specific assemblages become apparent. Although AMF diversity is well represented by Glomeromycota, ECM taxa appear to predominate overall when considering the combined richness of all families performing this symbiotic mode.

At the order and family levels, Glomerales and Glomeraceae consistently represented a major component of the communities, underscoring the pervasive role of AMF across *igapó* forests. Within the Agaricales, Entolomataceae (ECM) ranked as the second most ASV‐rich family, with *Entoloma* as the most prominent genera. The order Trechisporales also contributed substantially to ECM diversity, with richness concentrated in Hydnodontaceae and *Trechispora* as the most representative genus that inhabits the soil, but taxonomic information and DNA sequences of root and basidiomata fungi are still poorly reported in Brazilian biomes (Vanégas‐Léon et al. 2019).

Likely, the fungal community composition reflects the distribution of host trees and the influence of environmental filters (Junk et al. 1989; Scudeller and Villarrúbia 2018). In general, the floristic variation in *igapós* along the Rio Negro basin is high, although with most trees concentrated in a few families, such as Fabaceae, Lecythidaceae, and Sapotaceae, with some abundant species, such as *Tachigali* sp., *Pouteria elegans* and *Eschweilera albiflora* (Ferreira and Stohlgren 1999). Flood duration impacts plant diversity and distribution patterns, resulting in unique floristic compositions between low, medium, and high *igapós*. Comparisons between the *igapós* of the Jaú/Tarumã and Uatumã/Abacate Rivers show different tree assemblages at distinct topographic levels (Ferreira 2000; Ferreira and Stohlgren 1999; Wittmann et al. 2010; Lopes et al. 2014; Lobo et al. 2019). Floristic differences are attributed to environmental filters such as soil and flood regimes (Scudeller and Villarrúbia 2018; Lopes et al. 2014). Altogether, these patterns suggest that mycorrhizal assemblages in Amazonian *igapós* are structured by the interplay of flood regime, host identity, and basin‐specific environmental filters. Either way, our results suggest that Amazonian *igapós* maintain exceptional mycorrhizal diversity, particularly of ECM fungi, surpassing most other Neotropical ecosystems (Vasco‐Palacios et al. 2020; Corrales et al. 2022). This may include forests flooded by nutrient‐rich white water (várzea), which exhibit lower ECM fungi diversity, possibly due to greater nutrient availability (Bredin et al. 2021). These patterns underscore how flood regimes interact with local environmental filters and topography to shape complex mycorrhizal assemblages in these unique floodplain forests.

Flood‐level topography explained a smaller fraction of compositional variance than locality, yet its effects were consistent and ecologically interpretable. Flood levels integrate multiple environmental constraints relevant to fungi, including oxygen availability, redox conditions, and host turnover along the inundation gradient (Wittmann and Parolin 2005; Meyer et al. 2010; Householder et al. 2021, 2024). The taxonomic summaries suggest that flood regime modulates community composition within basins by shifting the relative representation of taxa already present in the regional species pool, rather than by generating entirely distinct assemblages. This supports a two‐tier assembly framework in which basin‐scale environmental context defines the available fungal pool, while flood‐level topography acts as a filter shaping relative abundances within that pool.

Differences in the balance between ECM‐ and AMF‐associated lineages across basins further reinforce this composition‐centered interpretation. While AMF lineages remained prominent across all sites, ECM‐associated taxa formed a substantial and sometimes dominant component of *igapó* root communities. The relative prominence of ECM‐associated families in some basins, particularly RDS Uatumã, indicates that ectomycorrhizal fungi are not marginal in these blackwater flooded forests, challenging earlier assumptions that ECM are rare or functionally unimportant in tropical floodplain systems. Rather than responding uniformly to flooding, mycorrhizal symbiotic modes appear to be differentially favored depending on basin‐specific host composition and environmental conditions.

These multivariate patterns demonstrate that *igapó* mycorrhizal communities are structured by meaningful site‐level differentiation expressed through shifts in dominance, compositional turnover at finer taxonomic ranks, and variation in community heterogeneity among basins. Although samples broadly overlap, these patterns indicate that Amazonian blackwater flooded forests host distinct belowground symbiotic assemblages shaped by hydrology, basin connectivity, and host tree composition.

Consistent with river‐network connectivity (Vannote et al. 1980), the Rio Negro links the Jaú and Cuieiras basins, exhibiting broadly similar assemblages, characterized by strong representation of AMF lineages, particularly Glomeraceae, alongside consistently present ECM‐associated families such as Entolomataceae and additional ECM taxa at lower relative abundances. In contrast, the more isolated Abacate River (RDS Uatumã) forms a distinct assemblage characterized by higher abundances of unidentified ECM lineages and a shift in the relative balance between ECM‐ and AMF‐associated taxa, with Entolomataceae exceeding Glomeraceae in relative abundance. This pattern suggests that basin context influences not only which fungal taxa occur but also their relative dominance within communities. The elevated proportion of unidentified lineages at RDS Uatumã likely reflects both genuine compositional distinctness and limitations of current reference databases for Amazonian root‐associated fungi, emphasizing the need for improved taxonomic coverage in the region.

### 4.4 ECM Diversity

The observed high diversity of ECM fungi and their compositional variability across topographies and rivers, in particular compared to AMF, challenges the long‐held assumption that ECM are rare in tropical forests and largely restricted to a small set of tree families. Instead, our data add to recent evidence of unexpectedly high ECM diversity in the Neotropics, including novel associations with families not previously recognized as ECM hosts (e.g., Lecythidaceae, Sapotaceae, Humiriaceae). Moreover, our results show that dominant trees are not always the main ECM hosts; in contrast, species of lower abundance often harbored disproportionately high ECM diversity.

In contrast to Singer and Araújo (1979) who suggested that low‐level *igapós* do not favor ECM due to oxygen limitation, our data indicate that the opposite may occur: in the Abacate River (RDS Uatumã), the highest ECM diversity occurs in low‐level *igapós*, which remain flooded for longer periods. An intermediate pattern may also occur as we found in the Cuieiras River. The reasons remain elusive, but may be related to plant adaptations such as well‐developed root aerenchyma that ensure minimum oxygen concentration required by the ECM fungi. Additionally, fungi adaptations, such as membrane proteins such as aquaporins may aid in the suberization of the root sheath in ectomycorrhizas in waterlogged soils (Khan 1993; Haase and Rätsch 2010; Meyer et al. 2010; Dietz et al. 2011; Johnson 2018). Alternatively, the mycelium range of fungal individuals may reach out of the flooded areas to the more oxygen‐rich soil of the *igapó* margin or local terra‐firme spots, assuring the oxygen supply. In any case, these results reinforce that the seasonal flood of *igapós* does not constitute a principal barrier for the fungi survival in these soils, particularly the mycorrhizae among the rhizospheres.

ECM‐associated orders such as Agaricales and Trechisporales, and families including Entolomataceae, Russulaceae, Thelephoraceae, and Hydnodontaceae contributed substantially to compositional differences among sites. These patterns were maintained at the genus level, where a small number of abundant genera accounted for a large fraction of ASVs, accompanied by a long tail of low‐abundance lineages grouped as “Other” or “Unidentified,” a characteristic feature of metabarcoding datasets in understudied tropical systems.

## 5 Conclusion

Our study demonstrates that Amazonian *igapó* tree roots harbor highly diverse mycorrhizal communities whose composition is shaped by the interplay between flood regime, topography, locality, and host tree composition. Despite the strong environmental constraints imposed by prolonged flooding, *igapó* forests sustain unexpectedly high mycorrhizal diversity, particularly of ectomycorrhizal fungi, potentially exceeding that reported for many other Neotropical ecosystems. Fungal diversity peaks differed among basins, occurring in low‐level *igapós* of the Abacate River, high‐level *igapós* of the Jaú River, and mid‐level *igapós* of the Cuieiras River, underscoring the dominant role of basin‐scale environmental context over topography alone. Community differentiation was expressed primarily through shifts in taxonomic composition and relative dominance rather than complete species turnover, indicating that flood‐level topography acts as a secondary filter on basin‐specific species pools. The high richness and prevalence of ECM fungi challenge the long‐standing view that ectomycorrhizal symbioses are rare or marginal in tropical floodplain forests. With 307 ECM ASVs detected, comparable to the 406 previously documented ECM lineages across the entire Neotropics, our results identify *igapó* forests as previously unrecognized hotspots of mycorrhizal specialization. These findings highlight the resilience and adaptability of plant–fungal symbioses under seasonal flooding and emphasize the importance of conserving *igapó* ecosystems in the face of ongoing hydrological alteration and climate change.

## Author Contributions

**Maihyra Marina Pombo:** conceptualization (equal), data curation (equal), investigation (equal), methodology (equal), writing – original draft (equal), writing – review and editing (equal). **Camila Duarte Ritter:** formal analysis (equal), visualization (equal), writing – review and editing (equal). **Florian Wittmann:** conceptualization (equal), project administration (equal), supervision (equal). **Jadson José Souza de Oliveira:** conceptualization (equal), data curation (equal), supervision (equal), writing – review and editing (equal). **Maria Teresa Fernandez Piedade:** funding acquisition (equal). **Jochen Schongart:** funding acquisition (equal). **Alexander Zizka:** funding acquisition (equal), visualization (equal), writing – review and editing (equal).

## Funding

This study was supported by the Long‐Term Ecological Research Program PELD‐MAUA (Phases 3 and 4), funded by CNPq and FAPEAM, under the project titled “Demonstration sites of pristine and impacted oligotrophic wetland ecosystems in Central Amazonia: identifying trends and filling knowledge gaps” (CNPq Grant No. 441811/2020‐5, 446044/2024‐5; FAPEAM Grant No. 01.02.016301.02630/2022‐76), within the call CNPq/MCTI/CONFAP‐FAPs/PELD No. 21/2020. Long‐Term Ecological Research Program PELD‐MAUA (Phase 2), funded by CNPq and FAPEAM, under the project titled “Ecology and monitoring of vegetation in oligotrophic wetlands in Central Amazonia: anthropogenic impacts and implications for conservation in protected areas in the Negro and Uatumã river basins” within the call PELD/CNPq/CAPES/FAPs/BC/NEWTON FUND No. 441590/2016‐0, FAPEAM Grant No 015/2016. M.M.P. also acknowledges CAPES for a research fellowship.

## Conflicts of Interest

The authors declare no conflicts of interest.

## Supporting information


**Table S1:** Tree species sampled in Amazonian *igapó* plots across three study areas (RDS Uatumã, PARANA Jaú, and RDS Rio Negro), showing their relative abundance (Rel Abu.), calculated plot density (ind/m2), corresponding topographic position (Top), and the distribution status: dominant (D), rare (R) or intermediate (I). Density values were estimated by standardizing relative abundance by plot area.

**Figure S1:** Plot‐level variation in dominant families by locality and flood level.
**Figure S2:** Taxonomic composition across flood‐level topography.
**Figure S3:** Taxonomic composition across localities (river basins).

**Data S1:** ece373373‐sup‐0003‐Supinfo.zip.

## Data Availability

Genbank SUB16094505; All the required data are uploaded as Supporting Information.
