# Supplementary material for: Richness and Composition of Mycorrhizal Fungi Varies by Flood Level and River Basin in Oligotrophic Amazonian Seasonally Flooded Forests
Source: Ecol Evol. 2026 Apr 8;16(4):e73373. doi: 10.1002/ece3.73373 (PMC13062489; doi:10.1002/ece3.73373)
Supplement: Supplementary file 2 — Figure S1: Plot‐level variation in dominant families by locality and flood level. Figure S2: Taxonomic composition across flood‐level topography. Figure S3: Taxonomic composition across localities (river basins). [file ECE3-16-e73373-s003.docx]

Supplementary figures for: **Richness and composition of  mycorrhizal fungi varies by flood level and river basin in oligotrophic Amazonian seasonally flooded forests**

Maihyra Marina Pombo^1^, Camila Duarte Ritter^2,3*^, Florian Wittmann^1,4^, Jadson José Souza de Oliveira^1^ , Maria Teresa Fernandez Piedade^1^, Jochen Schongart^1^, Alexander Zizka^5*^

^1^Ecology, monitoring and sustainable use of wetlands group, Biodiversity Coordination, National Amazon Research Institute,  Avenida André Araújo, 2936, Manaus, Brazil.

^2^Juruá Institute, Manaus, Amazonas 69083-300, Brazil.

^3^National Amazon Research Institute,  Avenida André Araújo, 2936, Manaus, Brazil.

^4^Department of the Floodplain Institute, Karlsruhe Institute of Technology (KIT), Institute of Geography and Geoecology. Josef Street 1 D-76437 Rastatt Karlsruhe, Germany.

^5^Biodiversity of plants lab, Department of Biology, University of Marburg. Karl-von-Frisch-Straße 8, 35043 Marburg, Germany. ORCiD: 0000-0002-1680-9192

*Corresponding authors: [alexander.zizka@biologie.uni-marburg.de](mailto:alexander.zizka@biologie.uni-marburg.de); k[micaduarte@gmail.com](mailto:kmicaduarte@gmail.com)


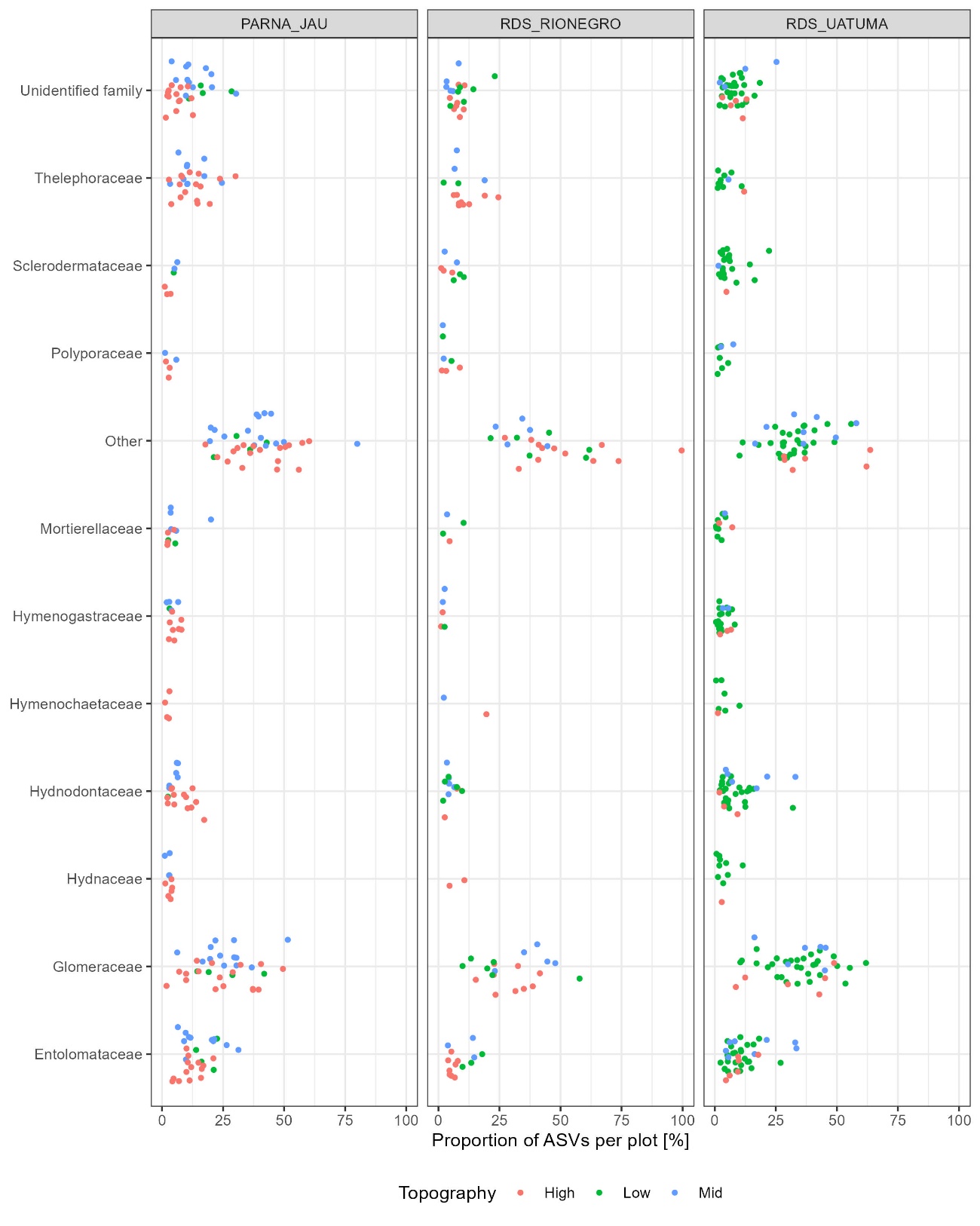


**Figure S1.** **Plot-level variation in dominant families by locality and flood level.** Proportion of ASVs per plot (%) for dominant fungal families across localities (PARNA Jaú, RDS Rio Negro, RDS Uatumã). Each point represents one plot; colours indicate flood-level topography (High, Mid, Low). Families correspond to those highlighted as dominant in the rank-based summaries; remaining families are pooled as **“Other”**, and ASVs without family-level assignment are shown as **“Unidentified family”**.


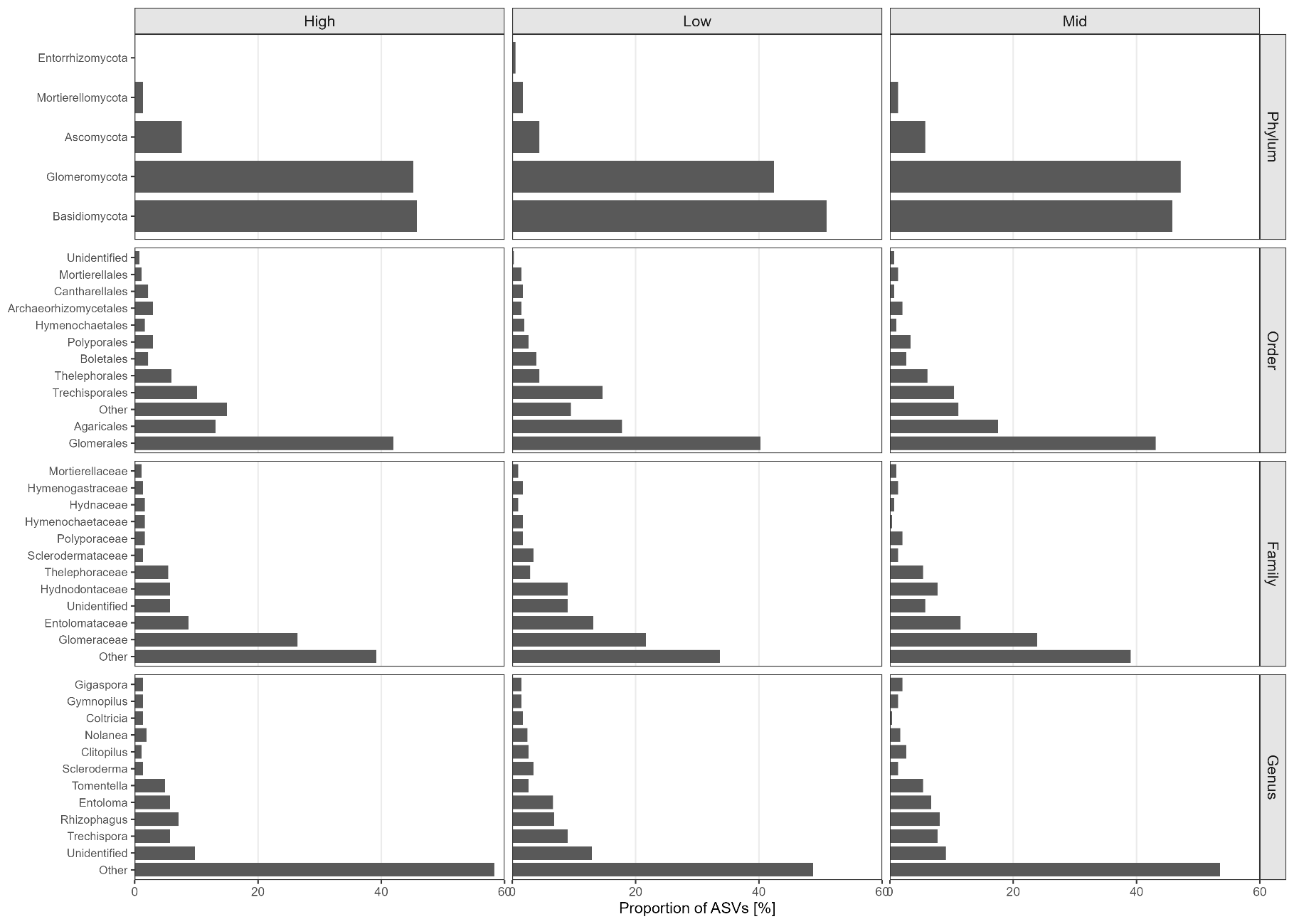


**Figure S2. Taxonomic composition across flood-level topography.** Relative contribution (proportion of ASVs, %) of the most abundant taxa at four taxonomic ranks (Phylum, Order, Family, Genus) across flood-level topographies (High, Mid, Low). Bars show the percentage of ASVs assigned to each taxon within a rank; taxa outside the top set are grouped as **“Other”**, and ASVs without assignment at that rank are shown as **“Unidentified”**.

**
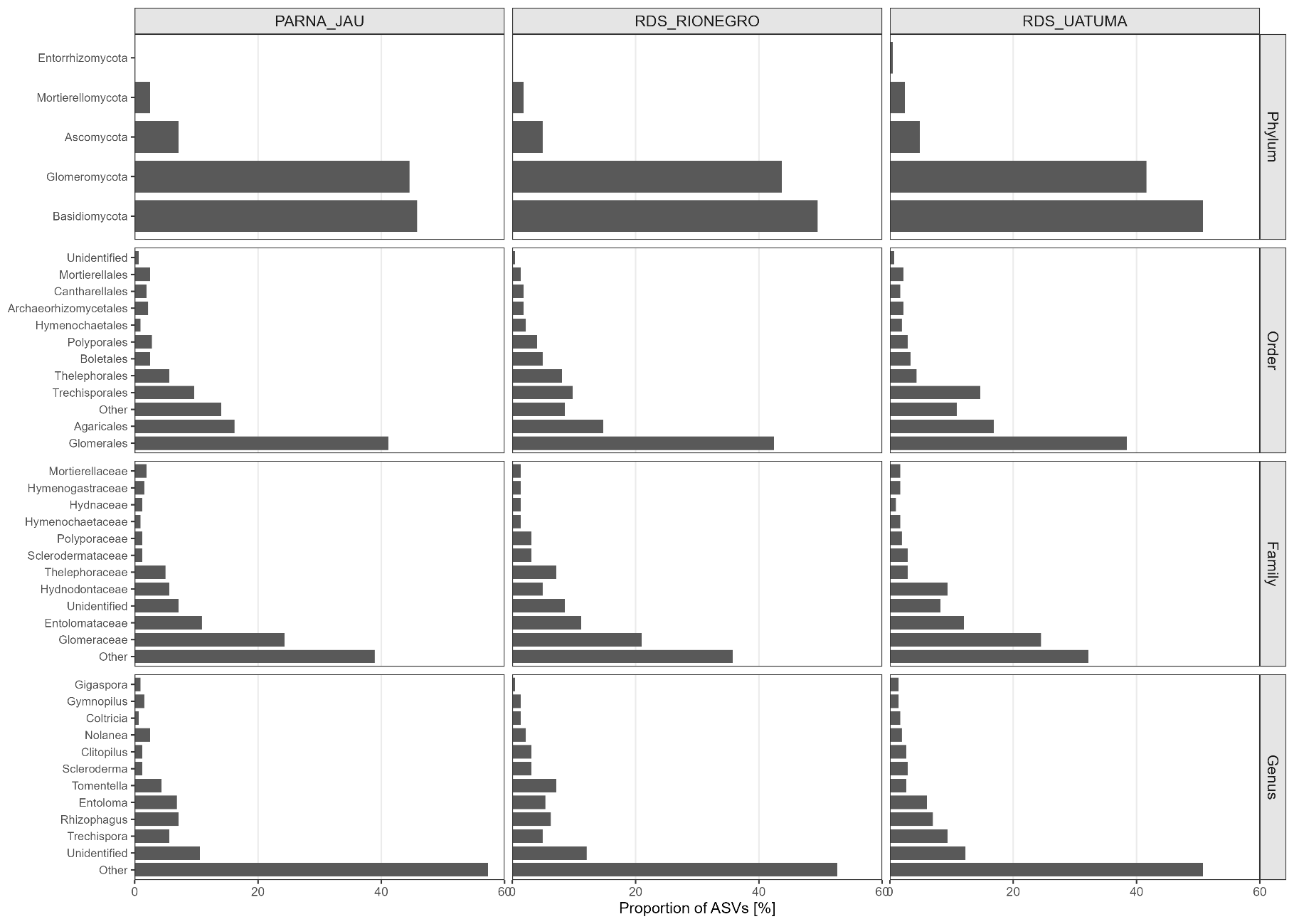
**

**Figure S3. Taxonomic composition across localities (river basins).** Relative contribution (proportion of ASVs, %) of the most abundant taxa at four taxonomic ranks (Phylum, Order, Family, Genus) across localities (PARNA Jaú, RDS Rio Negro, RDS Uatumã). Bars show the percentage of ASVs assigned to each taxon within a rank; taxa outside the top set are grouped as **“Other”**, and ASVs without assignment at that rank are shown as **“Unidentified”**.
